# A Novel Glutathione *S*-Transferase Gtt2 Class (VpGSTT2) Is Found in the Genome of the AHPND/EMS *Vibrio parahaemolyticus* Shrimp Pathogen

**DOI:** 10.3390/toxins13090664

**Published:** 2021-09-17

**Authors:** Ignacio Valenzuela-Chavira, David O. Corona-Martinez, Karina D. Garcia-Orozco, Melissa Beltran-Torres, Filiberto Sanchez-Lopez, Aldo A. Arvizu-Flores, Rocio Sugich-Miranda, Alonso A. Lopez-Zavala, Ramon E. Robles-Zepeda, Maria A. Islas-Osuna, Adrian Ochoa-Leyva, Michael D. Toney, Hugo Serrano-Posada, Rogerio R. Sotelo-Mundo

**Affiliations:** 1 Laboratorio de Estructura Biomolecular, Centro de Investigación en Alimentación y Desarrollo, A.C. (CIAD), Hermosillo 83304, Sonora, Mexico; ignaciovchavira@gmail.com (I.V.-C.); orozco@ciad.mx (K.D.G.-O.); 2 Departamento de Ciencias Químico-Biológicas, Universidad de Sonora, Hermosillo 83000, Sonora, Mexico; aldo.arvizu@unison.mx (A.A.A.-F.); rocio.sugich@unison.mx (R.S.-M.); Alexis.lopez@unison.mx (A.A.L.-Z.); robles.zepeda@unison.mx (R.E.R.-Z.); 3 Departamento de Ciencias de la Salud, Universidad de Sonora, Cd. Obregón 85040, Sonora, Mexico; david.corona@unison.mx; 4 Departamento de Investigación en Polímeros y Materiales, Universidad de Sonora, Hermosillo 83000, Sonora, Mexico; melissabt21@gmail.com; 5 Departamento de Microbiología Molecular, Instituto de Biotecnología (IBt), Universidad Nacional Autónoma de Mexico (UNAM), Cuernavaca 62210, Morelos, Mexico; fily@ibt.unam.mx (F.S.-L.); adrian.ochoa@ibt.unam.mx (A.O.-L.); 6 Laboratorio de Genética Molecular de Plantas, Centro de Investigación en Alimentación y Desarrollo, A.C. (CIAD), Hermosillo 83304, Sonora, Mexico; islasosu@ciad.mx; 7 Department of Chemistry, The University of California, Davis, CA 95616, USA; mdtoney@ucdavis.edu; 8 Consejo Nacional de Ciencia y Tecnología, Laboratorio de Agrobiotecnología, Tecnoparque CLQ, Universidad de Colima, Colima 28629, Colima, Mexico

**Keywords:** glutathione s-transferase (GST), *Vibrio parahaemolyticus*, Gtt2 class, glutathione (GSH), kinetic isotope effect, crystal structure

## Abstract

Glutathione S-transferases are a family of detoxifying enzymes that catalyze the conjugation of reduced glutathione (GSH) with different xenobiotic compounds using either Ser, Tyr, or Cys as a primary catalytic residue. We identified a novel GST in the genome of the shrimp pathogen *V. parahaemolyticus* FIM- S1708^+^, a bacterial strain associated with Acute Hepatopancreatic Necrosis Disease (AHPND)/Early Mortality Syndrome (EMS) in cultured shrimp. This new GST class was named Gtt2. It has an atypical catalytic mechanism in which a water molecule instead of Ser, Tyr, or Cys activates the sulfhydryl group of GSH. The biochemical properties of Gtt2 from *Vibrio parahaemolyticus* (VpGSTT2) were characterized using kinetic and crystallographic methods. Recombinant VpGSTT2 was enzymatically active using GSH and CDNB as substrates, with a specific activity of 5.7 units/mg. Low affinity for substrates was demonstrated using both Michaelis–Menten kinetics and isothermal titration calorimetry. The crystal structure showed a canonical two-domain structure comprising a glutathione binding G-domain and a hydrophobic ligand H domain. A water molecule was hydrogen-bonded to residues Thr9 and Ser 11, as reported for the yeast Gtt2, suggesting a primary role in the reaction. Molecular docking showed that GSH could bind at the G-site in the vicinity of Ser11. G-site mutationsT9A and S11A were analyzed. S11A retained 30% activity, while T9A/S11A showed no detectable activity. VpGSTT2 was the first bacterial Gtt2 characterized, in which residues Ser11 and Thr9 coordinated a water molecule as part of a catalytic mechanism that was characteristic of yeast GTT2. The GTT2 family has been shown to provide protection against metal toxicity; in some cases, excess heavy metals appear in shrimp ponds presenting AHPND/EMS. Further studies may address whether GTT2 in *V. parahaemolyticus* pathogenic strains may provide a competitive advantage as a novel detoxification mechanism.

## 1. Introduction

*Vibrio parahaemolyticus* is a Gram-negative bacterium that is part of the microbiota of marine organisms. Most *Vibrio* strains are pathogenic to invertebrates only. The pathogenicity of *V. parahaemolyticus* to humans depends on genetic markers that encode proteins such as adhesins, thermostable direct hemolysin (*tdh*), TDH-related hemolysin (*trh*), and two type-III secretion systems: T3SS1 and T3SS2 [1]. Many *Vibrio* strains are part of the marine microbiota, with some environmental strains containing pathogenic markers [2].

A comparison of the genome of a shrimp-pathogenic strain of *V. parahaemolyticus* (FIM- S1708+) and an innocuous (FIM-S1392-) strain identified more than 400 ORF differences [3]. Included in these are novel glutathione S-transferases similar to the Gtt1 and Gtt2 genes that have been previously reported as exclusive to fungi, specifically *Saccharomyces cerevisiae* [4,5,6,7].

Glutathione S-transferases (GSTs, EC 2.5.1.18) are ubiquitous enzymes that participate in phase II of the cellular detoxification system. They exist as dimers, with a 22–30 kDa monomer molecular weight [8]. Each monomer comprises an N-terminal glutathione-binding domain with a fold similar to thioredoxin (G-site) and a C-terminal globular binding site for the hydrophobic substrates such as xenobiotics, known as the H site [9].

The GSTs are classified into mitochondrial, microsomal, or MAPEG families, and as members of the cytosolic class. The latter class is diverse. In bacteria, four main classes of GSTs (beta, chi, theta, and zeta) exist [10], with the beta class being the best characterized [11]. The theta and zeta classes are the most divergent GSTs, since they are also present in mammals and plants [12]. Some families perform additional functions besides detoxification: the theta GSTs from fac-ultative methylotrophic bacteria have dichloromethane (DCM) dehalogenase activity [13], while the zeta class has tetrachlorohydroquinone (TCHQ) dehalogenase activity [14]. The YfcG bacterial class can bind oxidized glutathione [4], while the YghU class can synthesize glutathionylspermidine [15].

The central step in GST catalysis is the nucleophilic attack by the GSH thiol on the xenobiotic, leading to a conjugated product [8]. The catalytic residue that facilitates deprotonation of the GSH thiol in the majority of GSTs is serine or tyrosine. However, the atypical GTT2 from yeast (ScGSTT2) instead uses a water molecule to activate the thiol. This conclusion is based on biochemical and structural data showing a water molecule coordinated by polar residues and located next to the GSH thiol [5].

We present a structural and biochemical study of a GST from *Vibrio parahaemolyticus* (VpGSTT2) that we postulate employs this atypical yeast GTT2 mechanism. The term “atypical mechanism” is used herein to denote the lack of a polar side-chain to act as a general base catalyst for GSH deprotonation. This work is the first study of a glutathione transferase class Gtt2 from bacteria; its biochemical and structural characterization is relevant to evolution and metagenomic considerations.

## 2. Results

### 2.1. Phylogenetic and Sequence Analysis of GSTs

**Figure 1:**
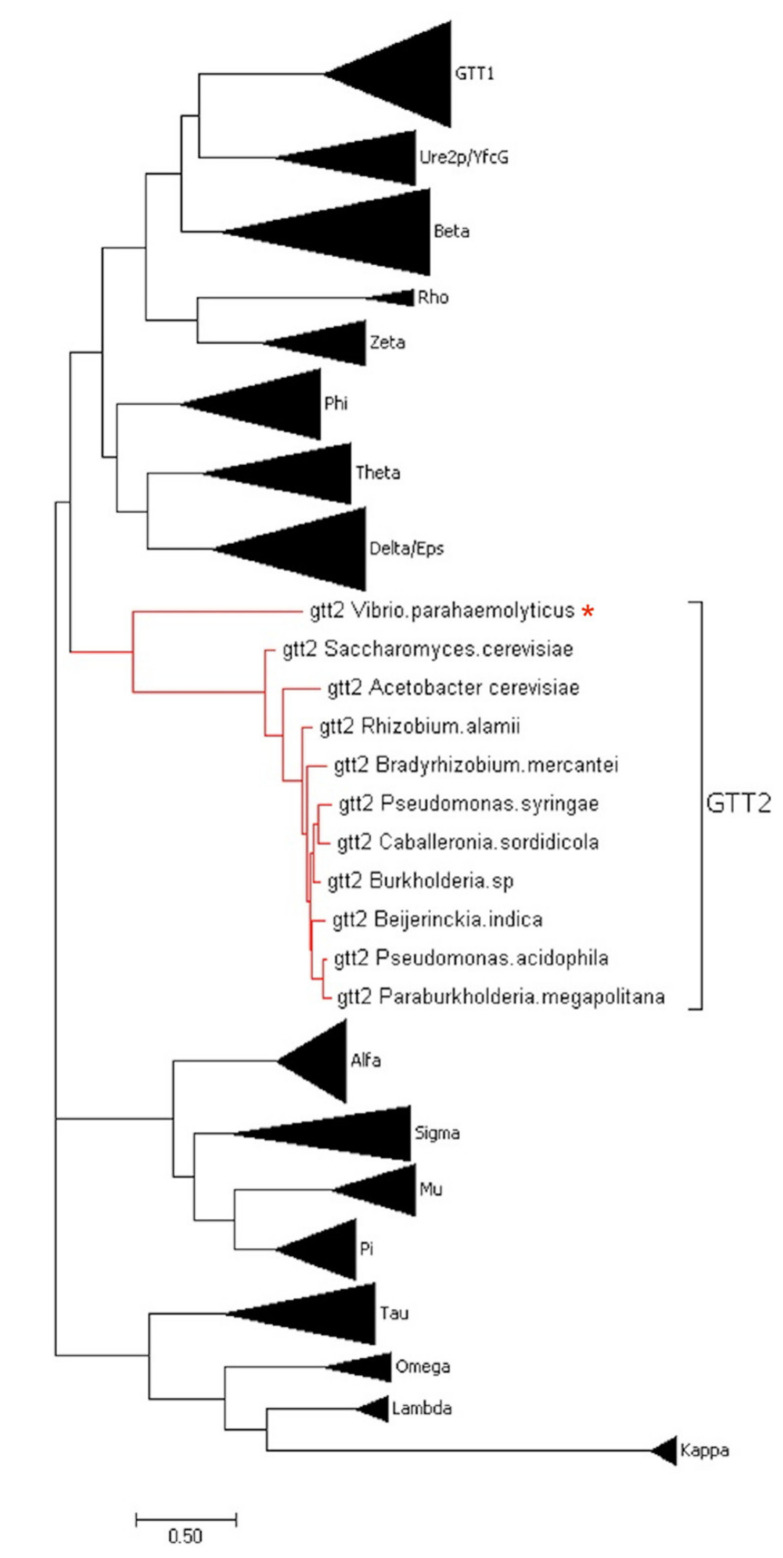
Phylogenetic tree with reported classed of GST; those belonging to the Gtt2 classes are highlighted in red. VpGSTT2 corresponds in the phylogenetic tree to Gtt2 Saccharomyces cerevisiae (labeled with a red asterisk). The amino acid sequences used were obtained from [11].

To obtain insights into the novel VpGSTT2 sequence features, a phylogenetic tree was constructed with the different GST classes (Figure 1). The VpGSTT2 is a Gtt2 member, which appears as an independent clade. Based on this result, we examined the VpGSTT2 active site amino acids for hints as to whether this enzyme uses the atypical catalytic mechanism reported for the Gtt2 class.

**Figure 2:**
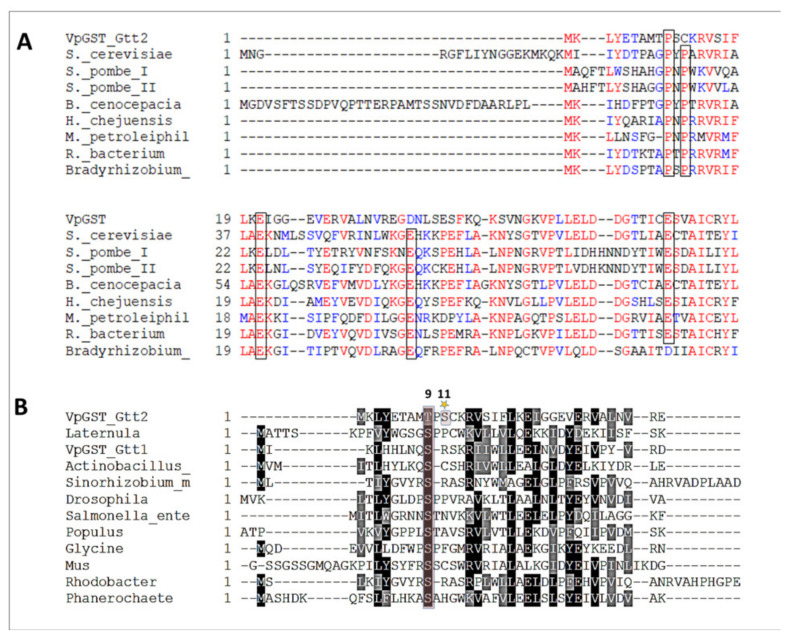
Sequence alignments. (**A**) GST sequences using the atypical catalytic mechanism. The black boxes represent mechanistically conserved residues. (**B**) GST sequences that used Ser9 as a catalytic residue. The shaded box highlights the conserved Ser, which in VpGSTT2 was a Thr, with Ser11 (highlighted with a star) two residues further toward the C-terminus. The sequences were obtained from [5].

Figure 2A shows a protein sequence alignment with GSTs that use the atypical mechanism, including ScGSTT2. VpGSTT2 has some of the residues conserved for this class. Figure 2B presents an alignment of VpGSTT2 with a diverse set of GSTs that use serine as a catalytic residue. From the sequence alignment, the most likely nucleophile in VpGSTT2 would be T11.

### 2.2. Overexpression and Purification of VpGSTT2

**Figure 3:**
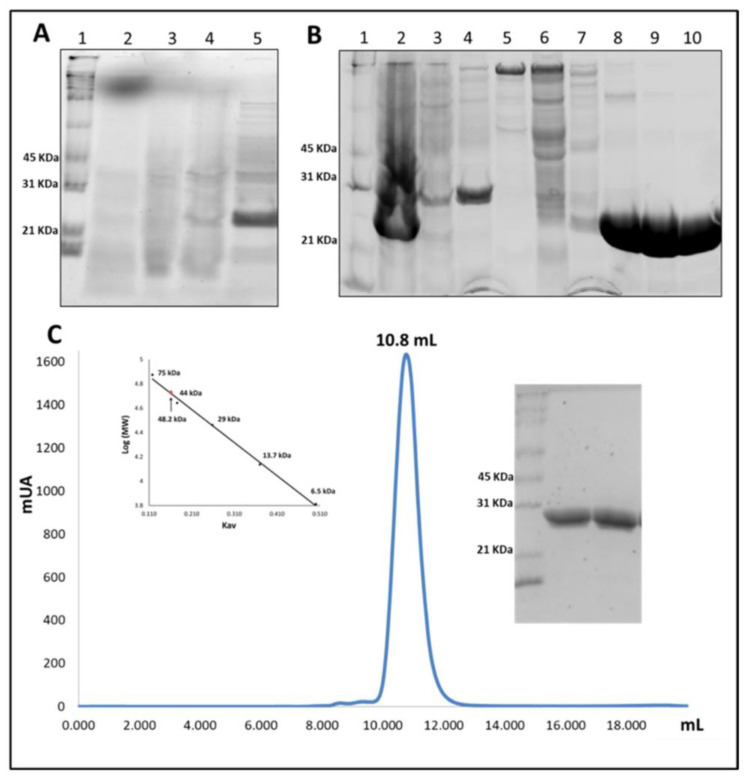
Purification of VpGSTT2. (**A**) SDS-PAGE of expression time points: lane 1 is the molecular weight marker (MWM); lanes 2–5 are samples from 0, 2, 4, and 24 h, respectively. The highest level of soluble VpGSTT2 (25 kDa) was found 24 h after induction. (**B**) SDS-PAGE IMAC purified fractions of VpGSTT2: lane 1, MWM; lane 2, clarified soluble cell extract; lanes 3–4, unbound protein; lanes 5–7, 75 mM imidazole eluate; lanes 8–10, 125 mM imidazole eluate containing VpGSTT2. (**C**) Gel filtration chromatogram showing VpGSTT2 eluting at 10.8 mL, corresponding to 48.2 kDa. SDS-PAGE (right) on samples obtained from the gel filtration experiment.

VpGSTT2 was expressed in soluble form after 24 h of IPTG induction. During the first hours (2, 4, and 6) of the experiment, low amounts of recombinant protein were observed by SDS-PAGE, but at 24 h, significant quantities of soluble VpGSTT2 were observed (Figure 3A). VpGSTT2 was purified by IMAC, as shown in Figure 3B. It eluted at ~150 mM imidazole. The molecular weight of VpGSTT2 (approximately 25 kDa per monomer) was that expected based on the DNA sequence. The yield of pure protein was ~45 mg/g of the bacterial pellet. Using size-exclusion chromatography, we confirmed that VpGSTT2 is a dimer with an approximate molecular weight of 48.2 kDa (see Figure 3C).

### 2.3. Activity Assay and Parameter Kinetics for VpGSTT2

Specific activity was measured using a fixed concentration of enzyme with 1 mM GSH and 1 mM CDNB, and was found to be 5.66 units/mg for wild-type VpGSTT2 and 0.27 units/mg for the S11A mutant. The double mutant T9A/S11A had no detectable activity (see Table 1). The specific activity for VpGSTT2 was within the range for those reported for other GSTs; the lowest known value was for *Aspergillus fumigatus* GST at 0.025 U [16]. Higher specific activities ranged from 304 to 1297 units/mg for human liver GST [17] and 440 units/mg for the shrimp *Litopenaeus vannamei* Mu-class [18]. The specific activity of VpGSTT2 was higher than that of *E. coli* GST (0.52 units/mg) [19]. Interestingly, the specific activity for VpGTT2 was almost nine times higher than yeast GTT2 [5] (Table 1). The catalytic efficiency of plant GSTs was generally higher than that of bacterial GSTs. For example, the specific activities of GSTs from *Nicotiana tabacum* and *Mangifera indica* were 48 and 44 units/mg, respectively [20,21].

**Figure 4:**
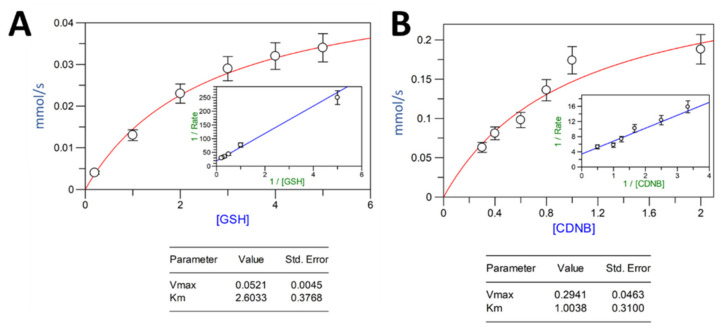
Michaelis–Menten plot for recombinant VpGSTT2: (**A**) GSH-varied; (**B**) CDNB-varied. Substrate concentrations are in mM.

The VpGSTT2 values shown in Table 1 are means calculated from three replicates. The kinetic parameters for VpGSTT2 were calculated for GSH and CDNB as substrates. The values obtained for *K_m_*, *V_max_*, *k_cat_*, and *k_ca_*_t_/*K_m_*, with GSH as the varied substrate, were 2.6 mM, 52.1 μM s^−1^, 8.7 s^−1^, and 3.3 mM^−1^ s^−1^, respectively (Table 2 and Figure 4). The values obtained from *K_m_, V_max_, k_cat_,* and *k_cat_/Km*, with CDNB as the varied substrate, were 1.0 mM, 294.1 μM s^−1^, 49 s^−1^, and 49 mM^−1^ s^−1^, respectively. The Michaelis–Menten and Lineweaver–Burk plots for each of the substrates are shown in Figure 4. We were unable to obtain reliable data for estimating the kinetic constants for the single or double mutant.

The Km value for GSH (2.6 mM) was consistent with those previously reported, with the lowest value of 0.05 mM for PvGSTU [23] and the highest of 2.5 mM for NsGSTT [24]. Most of the Km values reported for GSH were lower than 1 mM, so the relatively high Km of VpGSTT2 for GSH suggested a low affinity for this substrate (Table 2). For CDNB, the values for VpGSTT2 were similar to those previously reported.

### 2.4. Solvent Kinetic Isotope Effects: Proton Inventory

**Figure 5:**
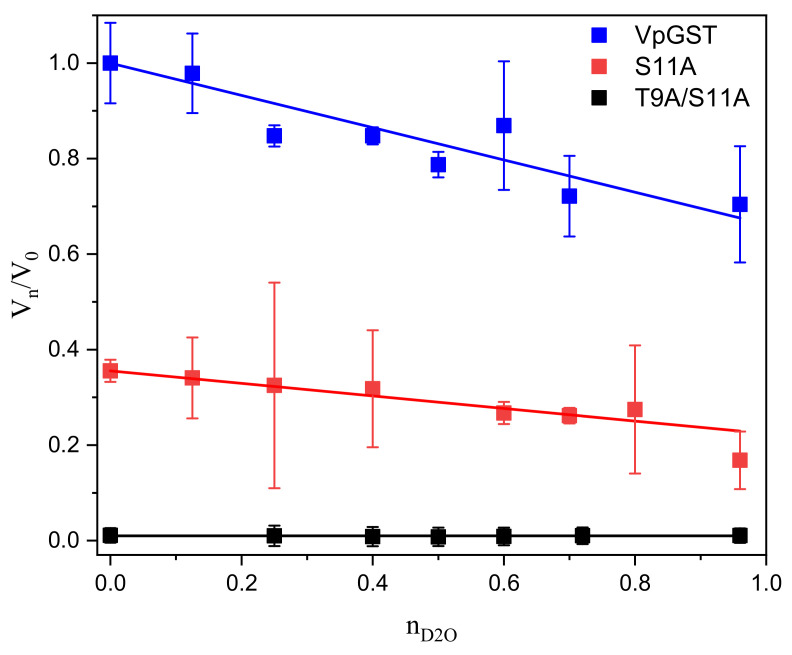
Proton inventory for native VpGSTT2 and mutants. The plot shows relative velocities at mole fractions n of D_2_O compared to that in pure H_2_O. Saturating CDNB (1.6 mM) and 1.0 mM GSH were used. The blue squares are the data for native VpGSTT2, and the red squares are for S11A. The lines represent the best fit to the Gross–Butler equation (V_n_/V_0_ = 1 − n + nϕ).

To probe if water participates in the activation of GSH and elucidates the number of protons in flight at the transition state, we utilized the proton inventory method [34]. We tested wild-type VpGSTT2 and mutant enzymes by measuring solvent effects on *k_cat_* (Figure 5). In all experiments, we observed linear behavior; and the data were fitted to the Gross–Butler equation, as shown in Figure 5. The linear dependence on the fraction of D_2_O in the solvent indicated that the solvent-sensitive transition state had a single proton in flight. The fractionation factor, ϕ, for the native and mutant (S11A) enzymes was 0.66 (±0.04) and 0.59 (±0.02), respectively, corresponding to kinetic isotope effects of ~1.6, within the expected range. The fractionation factors obtained with the proton inventory method correspond to the thiol group range (0.4–0.6) [35,36,37,38].

These values fell in the range (0.4–0.6) for fractionation factors of thiol groups (SH) [35,36,37], and agreed with the values reported for catalytic cysteines and thiols (or thiolates) [38,39,40]. The double mutant T9A/S11A was inactive and did not show any isotope effect, as predicted if the nucleophilic residues were absent.

### 2.5. GSH and GTS Binding Thermodynamics

**Figure 6:**
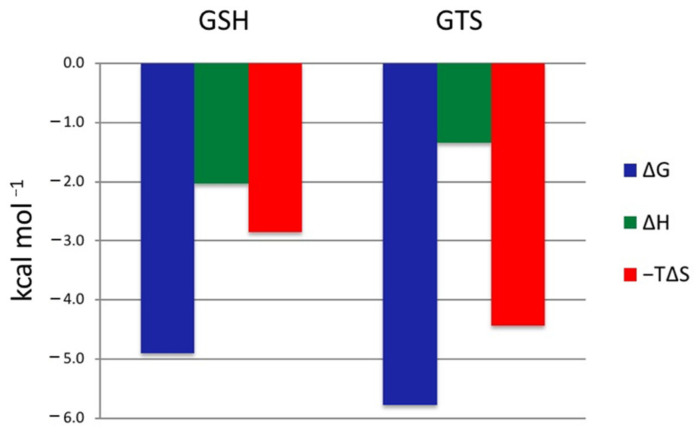
Freire diagram for the thermodynamic binding profile of VpGSTT2. The values of ΔG, ΔH, and −TΔS (kcal/mol) of the binding of VpGSTT2 with the GSH substrate (left) and the GTS inhibitor (right) are shown. Positive energy values are unfavorable for binding, while negative energy values reflect tighter binding to the enzyme.

ITC-based ligand titrations can directly determine the dissociation constant (K_d_) and the enthalpy (ΔH) for ligand binding, and thereby the Gibbs free energy (ΔG) and entropy (−TΔS) contributions to the binding. Figure 6 shows the Freire plot for the thermodynamic profiles for GSH and GTS binding. Both reactions were spontaneous, with ΔG values of −4.9 and −5.8 kcal/mol for GSH and GTS, respectively. The tighter binding GTS had ΔH and –TΔS values of −1.3 and −4.4 kcal/mol, respectively. Although the binding of GSH was spontaneous, the entropic contribution was larger than the enthalpic contribution (Table 3). Compared to the dissociation constant reported for other GSTs, VpGSTT2 had low affinity for GSH and GTS. We were unable to obtain reliable data for the single or double mutant.

### 2.6. Crystallographic Structure of VpGSTT2

**Figure 7:**
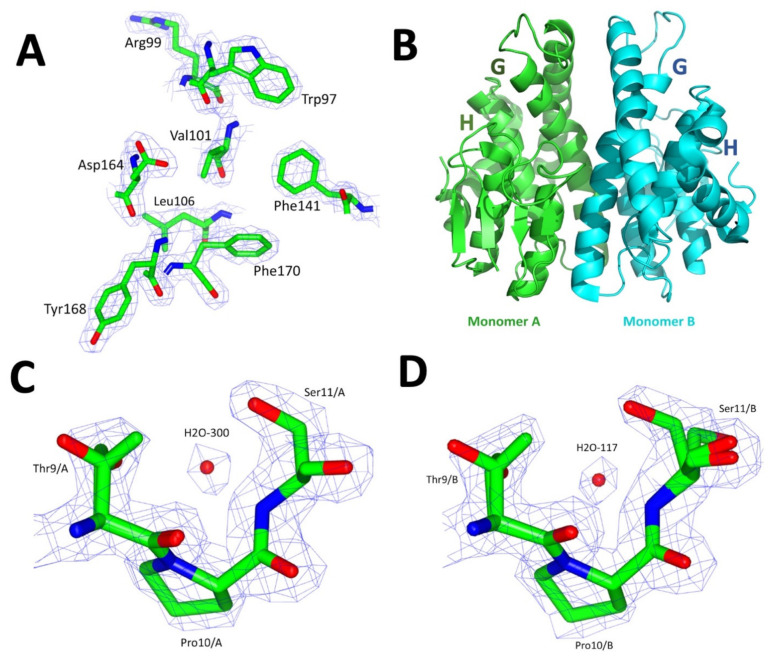
Crystallographic structure of VpGSTT2. (**A**) Representative electron-density maps (2Fo-Fc) for VpGSTT2 at 1.92 Å resolution. (**B**) Ribbon representation of the dimer of VpGSTT2 present in the asymmetric unit. (**C**) Residues (Ser11 and Thr9) that coordinated H2O-300 in monomer A. (**D**) Residues (Ser11 and Thr9) that coordinated the H2O-117 in monomer B. Electron density is displayed at 2σ in panels (**A**,**C**) and (**D**).

The crystal of VpGSTT2 had a monoclinic unit cell with space group P12_1_1, and the diffraction data of VpGSTT2-GTS were scaled and refined at a resolution of 1.92 Å (Table 4). After molecular replacement with a Phyre2-generated homology model monomer, a solution was obtained with a TFZ value of 74.4 and an LLG of 10,400. The final refined crystallographic structure had *R_work_/R_free_* values of 16.3%/20.8%, and 97% of the residues on the structure were on the most favored region of the Ramachandran plot (Table 4). The electron density for the representative residues of the crystallographic structure was observed, as shown in Figure 7A.

**Figure 8:**
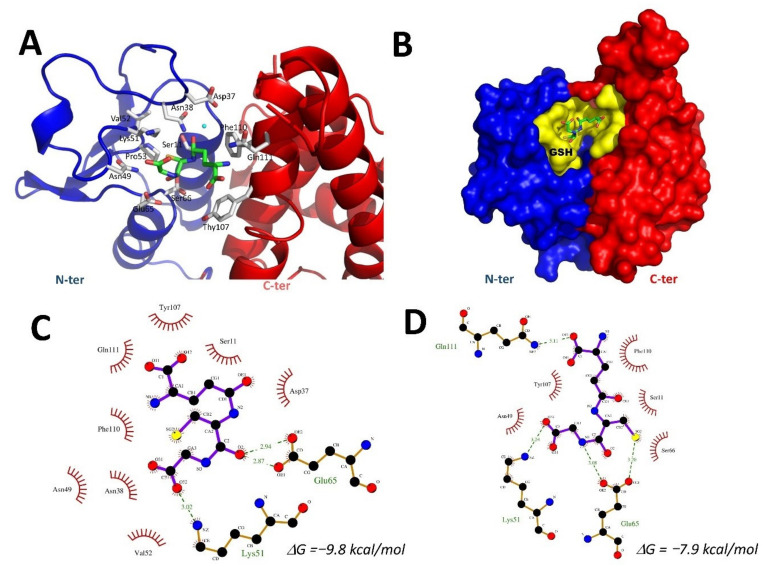
G-site of VpGSTT2. (**A**) VpGSTT2 residues of the G-site are shown as white sticks, the N-terminus in blue, the C-terminus in red, and H2O-300 as a blue sphere. (**B**) Surface representation of VpGSTT2, with the G-site in yellow and GSH shown in green. (**C**) GSH docking Ligplot diagram of pose #2, and (**D**) pose #134. GSH is shown with purple bonds, hydrogen-bonded residues are shown in brown, hydrogen-bonds are shown as dotted lines in green, and hydrophobic interactions are shown in red.

VpGSTT2 consists of two monomers per asymmetric unit. Each has the canonical GST topology, with a thioredoxin-like domain at the N-terminus and a globular C-terminal domain. The GSH-binding domain (residues 1–81) is called the G-site, while the hydrophobic substrates are bound at the H-site (residues 82–204) (Figure 7B and Figure 8B).

The RMSD between the two monomers in the asymmetric unit was 0.36 Å, with only minor differences in the N-terminal domain’s loops and β-sheet 2. Figure 7 shows a monomer of VpGSTT2 in both ribbon and surface representations, highlighting the G-site amino acids. The G-site is where catalysis occurred. VpGSTT2 crystallized only in the absence of ligands.

A structural alignment between VpGSTT2 (PDB: 7MIQ) and yeast apo ScGSTT2-GTS (PDB: 3ERG) (both belonging to the Gtt2 family) resulted in an RMSD of 1.2 Å, with 66 identical residues out of 216 (31%). These structural differences were found mainly in the loop between β-sheet strands 2 and 3 and the α-helix of the C-terminal domain.

Although VpGSTT2 is an apo structure, a crystallographic water molecule coordinated by the potential nucleophiles Thr 9 and Ser11 was observed. The electron density was clear for residue H2O-300 for monomer A (Figure 7C) and H2O-107 for monomer B (Figure 7D).

We identified structurally similar proteins to VpGSTT2 using the Dali algorithm to search the PDB [43], which found a *Marinobacter aquaeolei* VT8 GST-like protein (PDB 4N0V), the previously mentioned yeast GTT2 (PDB 3ERF) [5], and a novel GST from *Methylobacillus flagellatus* (PDB 4GLT) [44]. Sequence identities were in a range of 28–31%, and RMSD values of 1.9 to 2.3 Å for structural superpositions. Of these, only the yeast GTT2 has been biochemically characterized.

### 2.7. Docking of GSH into the VpGSTT2 Crystal Structure

Since the experimentally obtained VpGSTT2 lacked a ligand, we used SwissDock [45] to position the substrate into the active site. The docking was performed without the selection of the G-site (blind docking) for unbiased identification of the GSH-binding site. The algorithm positively located the substrate in the G-site of VpGSTT2 (PDB 7MIQ), where polar residues T9 and S11 were near the GSH thiol group (Figure 8A). Hydrogen bonding with Ser11 was not observed in the docking results. Three hydrogen bonds were found for poses 2 and 134 (Figure 8C,D); two hydrogen bonds were found with Glu65, and additional hydrogen bonds with Lys51 and Arg14 for poses 2 and 134.

In the apo-VpGSTT2, a loop containing residues 36–39 moved towards the G-site cavity, causing steric hindrance to GSH binding. Although the docking was not conclusive in predicting exact hydrogen bonds between water molecules and the GST thiol, it is important to emphasize that the G-site was identified, and that a crystallographic water molecule was present and coordinated by Thr9 and Ser11.

## 3. Discussion

Steady-state enzyme kinetics showed that VpGSTT2 is catalytically active as a glutathione transferase, while ITC showed that GSH binds to the protein, although with low affinity compared to other GSTs. Neither sequence alignments nor the crystallographic structure of VpGSTT2 clearly identified the thiol deprotonation catalyst at the G-site. Usually, serine, cysteine, or tyrosine performs this function, but the lack of ligands in the VpGSTT2 structure precluded the identification of the precise substrate-binding site, and thereby the catalytic residue.

An interesting feature of the apo-VpGSTT2 crystal structure is the presence of ordered water molecules (one per each monomer), similar to what was observed in yeast GTT2 (Figure 7C,D). These water molecules were coordinated by Thr9 and Ser11 via hydrogen bonds and were well positioned to deprotonate GSH. Therefore, we complemented the structural work with site-directed mutagenesis to probe the roles of Thr9 and Ser11 in the mechanism.

**Figure 9:**
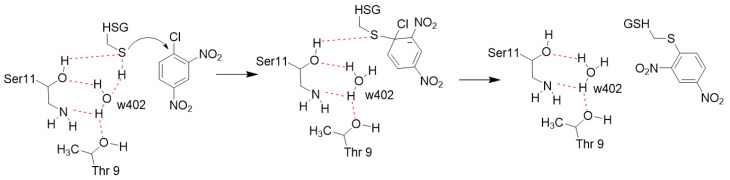
Proposed mechanism for Meisenheimer complex formation for VpGSTT2.

The S11A mutant retains 30% of wild-type activity, suggesting that the general base for deprotonating the glutathione sulfhydryl is likely to be the water molecule coordinated by Thr9 and Ser11. Therefore, the T9A/S11A double mutant was analyzed, since removing both residues to which the water molecule hydrogen bonds should eliminate it from the active site. The double mutant lacked detectable activity, which led us to conclude that water was the general base catalyst. Additionally, we hypothesized that Gtt2 class GSTs in general use a water molecule as the general base catalyst that activates GSH by deprotonation, increasing the nucleophilicity of the sulfhydryl such that it is able to form the Meisenheimer complex with CDNB (see Figure 9)

Proton inventories for wild-type and S11A demonstrated that a single proton was in flight in the transition state, and that this proton transfer was most consistent with thiol deprotonation, given the fractionation factors observed. These data precluded a pre-equilibrium proton transfer, obviating the need for forming the high-energy thiolate–hydronium ion pair in the active site. The ΔG values for ligand binding showed that VpGSTT2 had a lower affinity for GSH than other GSTs studied (Table 3). Most of the previous reports have unfavorable entropic components, compensated by strong enthalpic components, indicating important roles for hydrogen bonding and water reorganization in substrate binding. There not many examples of binding of GTS to GSTs in the literature. Nevertheless, comparing VpGSTT2 with the *Plasmodium falciparum* GST, we found that all components were favorable, and the higher dissociation constant correlated with an increase in the entropic part by an order of magnitude (–1.3 kcal mol^−1^ in *Vibrio* vs. –12.8 kcal mol^−1^ in the *Plasmodium*).

## 4. Materials and Methods

### 4.1. Overexpression and Purification of VpGSTT2

The sequence encoding VpGSTT2 was found on the genome of an environmental AHPND-strain FIM- S1708^+^ that was previously reported [3]. This sequence is present in the genome of some pathogenic strains. For this strain, the VpGSTT2 gene was deposited in GenBank under the accession code WP_025817526. Overexpression of recombinant VpGSTT2 was done using *E. coli* BL21(DE3) Gold. Bacteria were transformed with a construct based on vector pET11a (Novagen) and a synthetic linear DNA (gBlock, IDT) containing the coding region of the VpGSTT2 gene optimized for expression in *E. coli*. It contained a cut site for PreScission protease, and a 6xHis tag at the C-terminus [46]. The protein encoded in the construct has a theoretical molecular weight of 25 kDa.

The transformed bacteria were grown in LB broth with ampicillin (100 mg/mL) to OD_600_ 0.6 at 37 °C, followed by cooling the culture to 16 °C and addition of 0.4 mM IPTG. Bacteria were further incubated on an orbital shaker at 200 rpm for 24 h at 25 °C [47]. The broth was centrifuged at 8000× *g* at 4 °C in a Sorvall centrifuge for 15 min. One gram of the bacterial pellet was suspended in 5 mL of lysis buffer (0.1 M Tris-HCl, 0.5 M NaCl, 5 mM benzamidine, 5 mM dithiothreitol, 0.1 mg/mL lysozyme, and 1 mM phenylmethylsulphonyl fluoride, pH 7.5) followed by cell lysis with a sonicator (Branson Sonifier 450) for 15 s, followed by cooling for 2 min, repeating 3 times. The cell lysate was centrifuged at 20,000× *g* at 4 °C, and the soluble and insoluble fractions were analyzed by 12% SDS-PAGE stained with Coomassie Blue R-250 staining [47,48], or by fluorescence using 0.5% (*v/v*) of 2,2,2-trichloroethanol (TCE) in the polyacrylamide mix [49].

Purification was done by nickel IMAC using the His-tag [50,51]. Before passing the bacterial lysate through a 5 mL HisTrap HP column (GE Healthcare), it was filtered through a 0.45 mm pore-size filter. The column was pre-equilibrated with loading buffer (20 mM Tris-HCl, 0.5 M NaCl, pH 7.5), and after the injection of the lysate, was washed with eight volumes of purification buffer. The elution of VpGSTT2 was achieved via a linear gradient of 0 to 0.5 M of imidazole in the loading buffer. VpGSTT2 eluted at 150 mM imidazole in a final volume of 40 mL. The eluate was immediately dialyzed against 500 mL of 20 mM Tris-HCl pH 7.5 at 16 °C for 6 h. Fractions were analyzed in a silver-stained SDS-PAGE for a band at ~25 kDa corresponding to VpGSTT2 [47,48].

### 4.2. His-Tag Removal

After purifying VpGSTT2 by IMAC, it was extensively dialyzed against Tris-HCl pH 8, 150 mM NaCl, 10 mM EDTA, and 1 mM DTT. In the third exchange, 10 units of PreScission protease (GE Healthcare) were added to the contents of the dialysis membrane (12 kDa MW cutoff) for each mg of VpGSTT2 and incubated for 4 h. The His-tag was removed by dialysis, and the PreScission protease was removed by gel filtration chromatography. VpGSTT2 was dialyzed against Tris-HCl, 150 mM NaCl, pH 7.2. The quaternary structure of VpGSTT2 was analyzed by size-exclusion chromatography performed using a Superdex 75 HR 10/300 GL (GE Healthcare) column in an Äkta Pure instrument (GE Healthcare). The column was calibrated with molecular-weight standards: conalbumin (75 kDa), ovalbumin (44 kDa), carbonic anhydrase (29 kDa), ribonuclease A (13.7 kDa), and aprotinin (6.5 kDa) [47]. Protein concentration was determined using the bicinchoninic acid (BCA) method, with bovine serum albumin (1 mg/mL) as the standard reference.

### 4.3. Enzymatic Activity and Kinetic Parameters

To measure the activity of VpGSTT2, we used the method of [52], in which the conjugation of reduced glutathione (GSH) with the xenobiotic 1-chloro-2,4-dinitrobenzene (CDNB) leads to the formation of a 2,4-dinitrophenyl-S-conjugate absorbing at 340 nm (ε = 9600 M^−1^ cm^−1^) [52]. VpGSTT2 was dialyzed against the activity buffer containing 0.1 M NaH_2_PO_4_, pH 6.5. The reaction mixture was made in a 1 mL quartz cuvette with the activity buffer, 1 mM GSH, 1 mM CDNB, and 0.2 mg VpGSTT2. The reaction was monitored for 5 min at 340 nm in a Cary 50 Bio UV–vis spectrophotometer (Varian) at 25 C.

To determine the appropriate substrate concentrations for the GST assay, several reactions at different fixed concentrations of GSH (varying the CDNB concentration) were performed. All the experiments were performed by applying the initial rates method. In this method, the reaction was followed for 1 or 2% of completion [53]. A concentration of 0.5 mM GSH was optimal.

The reaction velocities for the calculation of *K_m_* and *V_max_* for the native and mutant enzymes were measured using a Hewlett-Packard 8453 diode array spectrophotometer by monitoring the GSH reaction with CDNB at 340 nm and room temperature. All experiments were performed with a blank reaction to subtract the nonenzymatic reaction rate between GSH and CDNB substrates. All measurements were made in triplicate. The *K_m_* and *V_max_* for the enzyme with GSH as substrate were obtained through varying the concentrations of GSH (0.04–5 mM) with constant CDNB concentration (0.8 mM). The values of *K_m_* and *V_max_* for CDNB were obtained by varying the concentration of CDNB (0.3–2 mM) with a constant level of GSH (0.5 mM). The Michaelis–Menten kinetic constants were calculated using a nonlinear regression fitting model with GraFit 7.0 (Erithacus Software).

### 4.4. Solvent Kinetic Isotope Effect: Proton Inventory

The proton inventory experiment was performed in H_2_O/D_2_O mixtures with phosphate buffer at pH 6.5. CDNB was used at saturating concentration (1.6 mM), and GSH was also close to saturation (1.0 mM). The deuterium mole fraction varied from 0 to 0.96. All enzymes, wild-type, and mutants were dissolved in H_2_O. The rest of the reactants were prepared in either D_2_O or H_2_O to ensure the required D_2_O/H_2_O ratio mixtures [54]. All assays were carried out in triplicate using 150 μg/mL of protein.

A plot of the measured kinetic solvent isotope effect on initial velocities (V_n_/V_0_) versus the deuterium mole fraction (n) was fitted to the Gross–Butler equation [34,35,55,56] using Origin 2018 software:

V_n_/V_0_ = 1 − n + nϕ (1)

### 4.5. Isothermal Titration Calorimetry

Microcalorimetric titrations were carried out using a MicroCal VP-ITC isothermal titration calorimeter at 298.15 K. The concentration of the enzyme (VpGSTT2) in the cell was 0.1739 mM in sodium phosphate buffer (50 mM) for a pH of 7.0. In the syringe, the concentration of the ligands (glutathione sulfonic acid GTS or GSH) was 10 mM in the same buffer as the enzyme. The experiment consisted of 31 injections: the first was 2 μL, and the rest were an 8 μL volume of GTS solution with a spacing time of 480 s between injections, a stirrer speed of 264 rpm, a filter period of 2 s, and a reference power of 10 μJ s^−1^ [4]. The raw data were corrected by subtracting the heat of dilution. The titration curve was analyzed using the AFFINIMETER software [57]. The solutions were previously degassed in a vacuum chamber for approximately 25 min.

### 4.6. Structure Determination

For crystallization trials, VpGSTT2 was extensively dialyzed against 20 mM Tris–HCl, pH 7.5, and concentrated with a 10 kDa ultrafiltration membrane (Amicon, Millipore, Burlington, MA, USA) to 10 mg/mL. Crystallization experiments were prepared using the hanging drop method in VDX48 plates (Hampton Research). The hanging drop method was used to improve the crystal size. We used the #66 solution from the Index crystallization kit (Hampton Research). That condition was optimized, and the best reservoir solution was 0.2 M ammonium sulfate, 0.1 M Bis-tris buffer pH 6.5, and 30% *w/v* polyethylene glycol 3350. Each drop consisted of a 2 μL reservoir solution and 2 μL protein sample (at 15 mg/mL), with 150 μL precipitant solution in the well. Subsequently, the plates were stored at 16 °C for three weeks. Crystal microseeding was used to improve morphology and size [30]. Crystals obtained from condition #66 from the Index kit were crushed and mixed with a micropipette and diluted 1:100 with mother liquor. One μL was used to seed the microbatch experiments to improve morphology.

The crystals of VpGSTT2 were diffracted at the Stanford Synchrotron Radiation Laboratory (SSRL) in the beamline 14-1 (λ = 1.181 Å). The crystals were soaked with the cryoprotection solution containing the crystallization solution with 25% *w/v* polyethylene glycol 400 and cooled with a liquid nitrogen stream at 100 K for the data collection [58,59].

The data were integrated and reduced with XDS [60], and processed in CCP4 with Combat, Pointless, and Scala [58,61,62,63]. Molecular replacement was performed in Phenix [64] with Phaser MR [65], using as the search model a homology model monomer obtained by Phyre2 [66] based on the coordinates of a GST structure of *Saccharomyces cerevisiae* class Gtt2 (Chain A from PDB: 3ERF) [5]. For the refinement of the structure, Phenix AutoBuild [67] and Polder omit maps were used, in addition to manual processing in COOT [68]. Finally, to be deposited in the PDB, the structure was validated with Molprobity [69]. The final crystal structure was deposited in the Protein Data Bank with the access reference 7MIQ.

### 4.7. Molecular Docking

The crystallographic structure of VpGSTT2 (PDB: 7MIQ) was considered as the receptor. The coordinates of the GSH ligand were obtained in PubChem [70], and were converted to the Mol2 format using OpenBabel [71]. Docking was performed with SwissDock [45] to predict the G-site in the VpGSTT2 crystal structure (PDB 7MIQ). SwissDock is based on an automatic coupling, with scripts to prepare the protein and ligands, estimating their CHARMM energies. The program iterates to 15,000 poses, selecting those with the most favorable energies. The results were visualized with UCSF Chimera [72]. The final poses were chosen based on their ΔG, ligand orientation, hydrogen bridges, and the binding cavity. The images were processed with Pymol [73], and the ligplots diagrams (2D interaction) were made with Ligplus [74].

## 5. Conclusions

VpGSTT2 is a novel GST found in the genome of a shrimp-pathogenic *V. parahaemolyticus* strain [3] Gtt2 class GST. The amino acid sequence contains the cognate N-terminal thioredoxin G-site domain and the hydrophobic H-domain. However, it is divergent from classical GST families, and it clusters with others (Figure 1) with an atypical enzyme mechanism.

By producing recombinant VpGSTT2, we demonstrated that this enzyme is active and uses GSH and CDNB as substrates. Calorimetric ITC assays showed that VpGSTT2 binds the GSH substrate and the GTS analog, but with lower affinity than other GSTs.

The crystal structure was solved in the absence of ligands. However, a water molecule was coordinated by both active sites by Thr9 and Ser11 residues, in a similar manner as reported for the well-studied yeast ScGTT2 [5]. As an extension of the X-ray model and to confirm that the experimental model could accommodate the substrate, we docked the GST molecule into the crystal structure. The substrate was found in the vicinity of Thr9 and Ser11, although not within hydrogen-bonding distance of either residue. This might be expected, since the docking process is rigid and does not accommodate ligand-induced conformational changes in the protein. Single and double mutants of the polar residues that coordinated water molecules suggested their critical role in catalysis.

The study of bacterial GSTs is limited compared to that of eukaryotic enzymes, but recent work suggests that the novel GTT2 family has alternative roles besides glutathione conjugation. These include detoxifying metals such as cadmium and nickel. Whether or not that function occurs in VpGTT2 and if it confers an advantage to shrimp-pathogenic strains remain to be determined. Cadmium levels above 0.9 μg/L in AHPND-positive ponds have been reported [75], although metals are not a variable that has been thoroughly studied. Other reports in which a GTT2 conferred metal resistance were related to *Burkholderia* cadmium resistance in tomato [76,77] and to nickel [78].

The causative agent for the AHPND/EMS syndrome is the plasmid-encoded PirA/B toxins found in environmental *V. parahaemolyticus* isolates [79]. However, it is not known if other genes provide a competitive advantage to a shrimp-pathogenic strain. It is important to mention that GSTs have the capability to detoxify cyanotoxins such as microcystin via glutathione conjugation [80], and that there are antagonistic interactions between marine bacteria and *Vibrio* sp. [81]. Whether GTT2 is able to provide a factor that contributes to PirA/B toxicity or provides protection to the host strain in adverse environmental conditions warrants further investigation.

In conclusion, VpGTT2 may be one of the multiple factors that distinguish and provide an advantage besides the PirA/B toxins or protection against chemical or biological stress to the AHPND/EMS pathogenic *V. parahaemolyticus* strains. Intensive shrimp farming and environmental factors are to be considered in preventing diseases in aquaculture.

## Figures and Tables

**Table 1:** Specific activities of GST isozymes.

GST	Specific Activity (units/mg)	Reference
VpGSTT2	5.66	This work
VpGSTT2 S11A	0.27	This work
VpGSTT2 T9A/S11A	not detectable	This work
Human liver	304–1297	[17]
*Aedes aegiptis* Epsilon-class	2.7–20.8	[22]
*Lipopenaeus vannamei* Mu-class	440.12	[18]
*Aspergillus fumigatus*	0.025	[16]
*Nicotiana tabacum*	48.61	[20]
*Mangifera indicaSaccharomyces cerevisiae* GTT2	43.76 0.63	[21] [5]
*Escherichia coli*	0.52	[19]

**Table 2:** Kinetic parameters for GSH and CDNB. The VpGSTT2 values shown are means calculated from three replicates.

	GSH			CDNB			
Enzyme	*K*_m_ (mM)	*k*_cat_ (s^−1^)	*k*_cat_/*K*_m_ (mM^−1^ s^−1^)	*K*_m_ (mM)	*k*_cat_ (s^−1^)	*k*_cat_/*K*_m_ (mM^−1^ s^−1^)	Reference
VpGSTT2	2.60	8.7	3.3	1.00	49	49	This work
NlGST1-1	0.66	118	180	0.26	118	447	[25]
AgGST1-1	0.807	97.3	120.7	0.12	97.4	792	[26]
DmGST1-1	0.28	28.3	101	0.80	38.3	48	[27]
LcGSTT	0.51	11.8	23.2	0.15	9	60.1	[28]
NsGSTT	2.50	0.013	ND	3.11	0.015	ND	[24]
GmGSTU4	0.159	6.05	38	0.16	2.48	15.7	[29]
GmGSTU10	0.068	2.65	39	0.28	2.66	9.5	[30]
MiGSTU	0.69	89.52	129	0.79	68.49	86.51	[21]
ZmGSTU1	0.56	NR	NR	1.01	18.6	18.4	[31]
ZmGSTU2	1.72	NR	NR	0.12	34.5	300	[31]
PtGSTU22	0.56	1118.39	1997.12	1.72	574.6	334.07	[32]
PvGSTU2-2	0.05	10.84	217	0.86	21.2	24.7	[23]
PvGSTU1-1	0.17	0.08	0.47	ND	ND	ND	[23]
CsGSTU1	0.5	0.014	0.028	0.75	0.024	0.032	[33]
CsGSTU2	0.5	0.077	0.154	1.0	0.108	0.108	[33]

**Table 3:** Thermodynamic parameters of GST binding with different ligands.

GST	Kd (μM)	ΔG (kcal/mol)	ΔH (kcal/mol)	−TΔS (kcal/mol)	Reference
VpGSTT2-GSH	257.8	−4.9	−2.00	−2.9	This work
VpGSTT2-GTS	57.9	−5.8	−1.4	−4.4	This work
MiGSTU-GSH	5.2	−7.2	−26.4	19.2	[21]
MiGSTU-GSX	7.8	−6.9	−6.2	−0.71	[21]
hGSTP1-GSH	85.98	−5.53	−11.21	5.65	[41]
hGSTP1-GSX	1.23	−8.04	−16.13	8.04	[41]
SjGST-GSH	0.99	−4.9	−5.7	0.8	[41]
AtGST-GSX	22.7	−26.1	−5.2	−20.9	[9]
PfGST-GSH	140	−5.7	−11.8	6.1	[42]
PfGST-GTS	162	−7.1	−12.8	5.7	[42]

Vp, *Vibrio parahaemolyticus*; Mi, *Mangifera indica*; h, human; Sj, *Schistosoma japonicus*; At, *Arabidopsis thaliana*; Pf, *Plasmodium falciparum*.

**Table 4:** X-ray data collection and refinement statistics.

Parameters	VpGSTT2-Apo
Data Collection Statics	
Space Group	P21
Unit cell dimensions	
a, b, c (Å)	56.3 50.4 69.6
*α, β, γ* (degrees)	90.0 90.1 90.0
Resolution range (Å)	40.8–1.92
No. of reflections	152,812 (20,045)
No. of unique reflections	29,139 (3971)
Completeness (%)	98.8% (94.3)
*R_meas_* (%)	5.4 % (18)
CC_1/2_ (%)	99.8 % (94.3)
*I/σ(I)*	5.8 (2.1)
Multiplicity	5.2 (5.0)
Monomers per asymmetric unit	2
**Refinement statistics**	
Resolution range (Å)	40.8–1.92
*R_work_*/*R_free_* (%)	16.3/20.8
*Number of reflections*	29,106 (2668)
*Clash score*	5.75
Mean *B*-values (Å^2^)	
Protein	28.6
Ion/Ligand	36.5
Water	33.6
All atoms	29.1
Wilson plot	23.4
RMSD from ideal stereochemistry	
Bond lengths (Å)	0.01
Bond angles (degrees)	0.61
Coordinate error (Maximum-Likelihood Base)	0.21
Ramachandran plot (%)	
Most favored region	96.3
Additional allowed regions	2.3
Disallowed regions	1.4
**PDB code**	**7MIQ**

Values in parentheses are for the last resolution shell.

## Data Availability

Protein Data Bank (www.rcsb.org, accessed on 1 July 2021); access reference 7MIQ.
